# Differential Effects of Three Techniques for Hepatic Vascular Exclusion during Resection for Liver Cirrhosis on Hepatic Ischemia-Reperfusion Injury in Rats

**DOI:** 10.1155/2018/5309286

**Published:** 2018-01-08

**Authors:** Changjun Jia, Chaoliu Dai, Hailiang Wang, Yi Wan, Yunyu Qiao, Feng Xu, Songlin Peng, Yang Zhao, Chuang Zhao, Liang Zhao

**Affiliations:** ^1^Department of Hepatobiliary and Splenic Surgery, Shengjing Hospital of China Medical University, Shenyang, Liaoning 110004, China; ^2^Department of Hepatobiliary Surgery, Weihai Central Hospital, Weihai, Shandong 264400, China; ^3^Department of Breast and Thyroid Surgery, Affiliated Zhongshan Hospital of Dalian University, Dalian, Liaoning 116001, China; ^4^Department of Anal Disease, Shenyang Coloproctology Hospital, Shenyang, Liaoning 110000, China

## Abstract

**Background/Aims:**

Hepatic ischemia-reperfusion (I/R) injury is a serious concern during hepatic vascular occlusion. The objectives of this study were to assess effects of three techniques for hepatic vascular occlusion on I/R injury and to explore the underlying mechanisms.

**Methods:**

Liver cirrhotic rats had undertaken Pringle maneuver (PR), hemihepatic vascular occlusion (HH), or hepatic blood inflow occlusion without hemihepatic artery control (WH). Levels of tumor necrosis factor alpha (TNF-*α*), nuclear factor kappa B (NF-*κ*B), toll-like receptor 4 (TLR4), TIR-domain-containing adapter-inducing interferon-*β* (TRIF), and hemeoxygenase 1 (HMOX1) were assayed.

**Results:**

The histopathologic analysis displayed that liver harm was more prominent in the PR group, but similar in the HH and WH groups. The HH and WH groups responded to hepatic I/R inflammation similarly but better than the PR group. Mechanical studies suggested that TNF-*α*/NF-*κ*B signaling and TLR4/TRIF transduction pathways were associated with the differential effects. In addition, the HH and WH groups had significantly higher levels of hepatic HMOX1 (*P* < 0.05) than the PR group.

**Conclusions:**

HH and WH confer better preservation of liver function and protection than the Pringle maneuver in combating I/R injury. Upregulation of HMOX1 may lead to better protection and clinical outcomes after liver resection.

## 1. Introduction

Hepatocellular carcinoma (HCC) is one of the most common cancers, and mortality rates for HCC remain high across the world [1, 2]. It has been well documented that cirrhosis is present in the vast majority of HCC patients. Indeed, hepatitis B virus (HBV) infection is one of the main causes of liver cirrhosis in many Asian countries and Pacific islands, while in many developed countries, such as the United States, Australia, and most countries in West Europe, chronic hepatitis C virus (HCV) infection is identified as one of the major risk factors of liver cirrhosis [1–3]. Of the treatment options for HCC, partial liver resection (LR) with a low mortality rate has been suggested as an effective approach for patients with HCC [4]. Over the past decades, substantial progress has also been made and has resulted in techniques for surgical procedures of liver resection for HCC that include a number of novel methods of hepatic vascular occlusion, in which the inflow of blood to the liver is blocked by enhancing the systemic vascular resistance and reducing the cardiac output, thereby allowing for great reduction in blood loss during liver resection [5–7]. Each hepatic vascular occlusion technique is selected based on several factors, such as the presence of liver cirrhosis and other liver disease, tumor location, cardiovascular condition, and so on. Although liver resection with hepatic vascular occlusion has been successfully performed in HCC with hepatic cirrhosis, poor tolerance of the cirrhotic liver to ischemia in HCC patients has been observed, and hepatic ischemia-reperfusion (I/R) injury, as an important cause of liver damage, has become a serious concern and poses challenges to liver surgeons [6–13]. In fact, I/R injury is characterized by inflammation, apoptosis, and tissue damage resulting from restoration of blood supply after previous vascular occlusion during operative procedures, and chemokines and signaling pathways have been associated with I/R-induced adverse effects, in particular, liver inflammation and liver damage that occur after hepatic vascular occlusion during liver resection [14–17]. Reduction of the nuclear factor kappa B (NF-*κ*B) activity with an inhibitor has been shown to protect the organ from I/R injury [18, 19].

Until now, a direct comparison of the effects of three techniques for hepatic vascular inflow occlusion during resection for liver cirrhosis including the Pringle maneuver [5], hemihepatic vascular occlusion, and hepatic blood inflow occlusion without hemihepatic artery control on I/R injury and the underlying molecular mechanisms has not been well studied. Therefore, there is a need for comprehensive studies to understand the similarities and differences between the techniques and to assess the efficiency of different hepatic vascular occlusion techniques and understand the underlying molecular mechanisms. In the present study, liver cirrhosis was induced in male Wister rats by alcohol and CCl_4_, and the Pringle maneuver, hemihepatic vascular occlusion, and hepatic blood inflow occlusion without hemihepatic artery of resection in the cirrhotic liver were applied. Comparative yet comprehensive studies of the effects of the three techniques on liver damage and the underlying mechanisms were carried out in rats. Our study may provide a scientific basis on which an appropriate procedure for hepatic vascular occlusion in HCC patients with liver cirrhosis can be selected and ultimately improve clinical outcomes for patients with liver cirrhosis-associated HCC.

## 2. Materials and Methods

### 2.1. Animals

A total of 122 male Wister rats with a body weight of 250–300 g were obtained from the Experimental Animal Center of Shengjing Hospital affiliated to China Medical University and kept until animal experiments were performed. The experimental rats were maintained in the center following the animal care and research protocol, which was approved by the Animal Care and Use Committee of China Medical University and complied with guidelines for the use of laboratory animal issued and implemented by the Chinese government.

The male Wister rats were used to establish an animal model for liver cirrhosis induced by increasing concentrations of alcohol and CCl_4_ as reported previously [20]. In brief, the rats were fed a normal diet and drinking water containing alcohol (5% *V*/*V*) and injected subcutaneously with 0.3 mL/100 g of 40% CCl_4_ diluted 1 : 1 (*V*/*V*) in olive oil twice a week. After 4 weeks, the concentration of CCl_4_ was increased to 50% and diluted 1 : 1 (*V*/*V*) in olive oil once every 3 days for 3 weeks. The rats were further treated with CCl_4_ with a higher concentration of 60% diluted 1 : 1 (*V*/*V*) in olive oil once every 3 days for additional 5 weeks to produce male Wister rats with hepatic cirrhosis.

### 2.2. Surgical Procedures for Liver Cirrhosis in Rats

**Figure 1:**
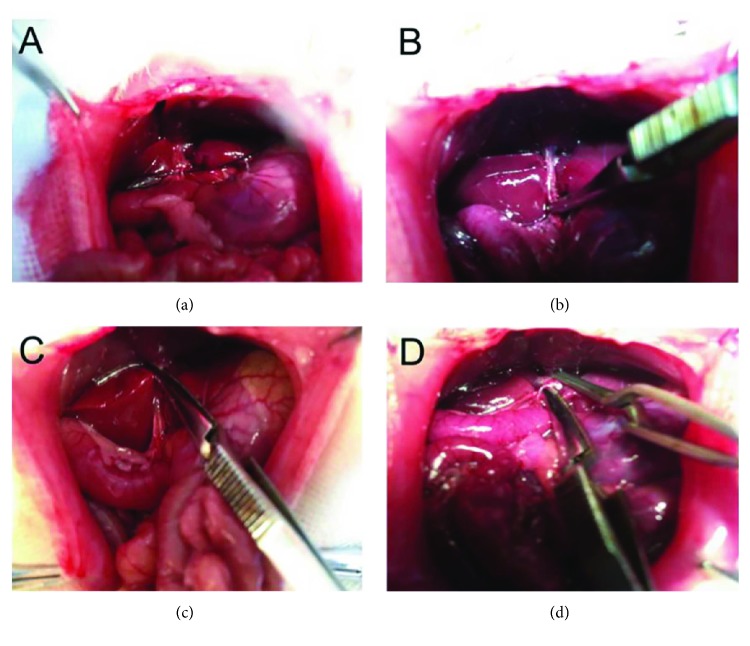
Graphical illustration of the three techniques for hepatic vascular occlusion during resection in the cirrhotic liver in rats. Hepatic cirrhosis was successfully induced in rats using a combination of alcohol and CCl_4_, and the rats were subjected to three different techniques for hepatic vascular occlusion during liver resection. (a) The entire hepatic portal system including the portal vein, artery, and biliary system was exposed; (b) in the Pringle maneuver group, the blood inflow to the entire liver was blocked by clamping across the hepatoduodenal ligament; (c) in the hemihepatic vascular occlusion group, the left hepatic portal vein and middle artery were occluded, allowing the normal blood supply to reach the right hemiliver; (d) in the hepatic blood inflow occlusion without hemihepatic artery control group, the portal vein was interrupted without interrupting the hepatic artery.

The rats with liver cirrhosis were randomly divided into three groups: Pringle maneuver group (PR group, *n* = 32), hemihepatic vascular occlusion group (HH group, *n* = 32), and hepatic blood inflow occlusion without hemihepatic artery control group (WH group, *n* = 32). The rats were anesthetized, and the abdomen was incised. As shown in Figure 1, in the PR group, the entire blood flow to the liver was interrupted by clamping across the hepatoduodenal ligament containing the hepatic artery, common bile duct, and portal vein, as first described by Pringle, a British surgeon, in 1908 [7]. To perform the liver resection in the HH group, the left portal vein and the middle hepatic artery were identified and blocked, which resulted in interruption of the blood supply of the left hepatic lobe as first introduced and applied during resection of the liver by Pringle et al. [5]. Since components of the hepatic portal system in humans and rats are distinct, hepatic blood inflow occlusion without hemihepatic artery control in the WH group was achieved by interrupting the left portal vein while the middle hepatic artery was retained. After 15 min of hepatic vascular occlusion in each group, the hepatic blood inflow was restored by reperfusion. According to reperfusion times of 1, 3, 6, and 24 h, each of the PR, HH, and WH groups was further divided into 4 subgroups (*n* = 8). After the liver surgical procedures were completed, the rats were sacrificed, and then liver tissues and the blood samples were collected properly and used for subsequent experiments.

### 2.3. Liver Histology

Liver samples were taken from the Wister rats that were treated with increasing concentrations of alcohol and CCl_4_ for induction of liver cirrhosis and fixed with 10% formalin for 24 h. Paraffin-embedded liver sections were routinely prepared in our laboratory. 5 *μ*m liver sections were mounted in glass slides, and hematoxylin-eosin (H&E) staining was subsequently conducted on each slide. Induction of liver cirrhosis in rats was evaluated by the histological changes in the liver, which was characterized mainly by disappearance of hepatic lobule structure, absence of central portal vein, and formation of false lobules and fibrous cord.

### 2.4. Immunohistochemistry

Immunohistochemistry (IHC) was performed to detect TIR-domain-containing adapter-inducing interferon-*β* (TRIF) protein expression in cells of the rat liver sections. Paraffin sections of the rat liver were dewaxed and hydrated. After washing with phosphate-buffered saline (PBS; 0.01 M, pH 7.5), the slides were incubated with rabbit anti-mouse polyclonal TRIF primary antibody (ZSGB-Bio, Beijing, China) overnight at 4°C and rinsed with PBS (0.01 M, pH 7.5) at room temperature (RT). A working solution containing a secondary antibody was added for incubation for 20 min at RT, and then the slides were washed with PBS (0.01 M, pH 7.5) at least three times. The slides were visualized with diaminobenzine (DAB), counterstained by hematoxylin, dehydrated, and mounted in a neutral resin mountant. Blue staining, as determination of TRIF protein expression, was displayed and examined using Nikon E800 microscope according to the manufacturer's instructions.

### 2.5. Biochemical Tests

Serum samples from the rats in each group were obtained and assayed for alanine aminotransferase (ALT) and aspartate aminotransferase (AST) levels using an ALT assay kit and AST assay kit, respectively, purchased from Jianchen Biotechnology Company (Nanjing, China).

### 2.6. Real-Time Quantitative RT-PCR

The primers used in this study were as follows: HMOX1-specific sense primer, 5′-AGGTCCTGAAGAAGATTGCG-3′ (20 bp), HMOX1-specific anti-sense primer, 5′-GATGCTCGGGAAGGTGAAAA-3′ (20 bp); GAPDH-specific sense primer, 5′-GCACCGTCAAGGCTGAGAAC-3′ (20 bp), GAPDH-specific anti-sense primer, 5′-ATGGTGGTGAAGACGCCAGT-3′ (20 bp). Real-time quantitative RT-PCR was carried out using the PrimeScript RT-PCR Kit in combination with quantitative PCR reagent from Clontech (Mountain View, CA, USA) following the manufacturer's instructions. Samples without template and reverse transcriptase were included as negative controls, and Ct values greater than 35 were considered as negative signals. Fold changes in HMOX1 expression were calculated by comparative Ct analysis after normalization to GAPDH expression in the same samples.

### 2.7. Western Blot Analysis

Western blot (WB) analysis was performed to determine protein levels of NF-*κ*B, Toll-like receptor 4 (TLR4), *β*-actin, and GAPDH in the rat liver tissues. In brief, total proteins were isolated from the liver tissues of the experimental rats. After 80 *μ*g of total proteins were separated on sodium dodecyl sulfate- (SDS) polyacrylamide gel electrophoresis (PAGE) gels, the gels were transferred onto polyvinylidene fluoride (PVDF) members, which were then blocked and incubated at 4°C overnight with primary antibodies including rabbit anit-mouse NF-*κ*B polyclonal antibody from Santa Cruz Biotechnology (Santa Cruz, CA, USA), mouse anti-rat TLR4 polyclonal antibody from Abcam (Cambridge, MA, USA), rabbit anit-mouse polyclonal *β*-actin antibody from ZSGB-Bio (Beijing, China), and GAPDH antibody from Abcam (Cambridge, MA, USA). The specific bands were visualized and analyzed with Quantity One imaging system from Bio-Rad (Hercules, CA, USA). The intensity of NF-*κ*B and TLR4 bands was normalized to *β*-actin or GAPDH.

### 2.8. Examination of Cytokine Levels by ELISA

Blood samples were obtained from each group of the rats. Levels of selected cytokines were measured using enzyme-linked immunosorbent assay (ELISA) kits (R&D Systems, Minneapolis, MN, USA) for tissue necrosis factor alpha (TNF-*α*) following the manufacturer's instructions.

### 2.9. Statistical Analysis

Statistical analysis was performed with IBM SPSS Statistics version 11.5 from SPSS Inc. (Chicago, IL, USA), and one-way analysis of variance (ANOVA) was used. Prior to the one-way ANOVA test, Levene's test for equality of variances was conducted. Either S-N-K test or Dunnett's T3 test was used to analyze differences between groups. *P* < 0.05 was considered statistically significant.

## 3. Results

### 3.1. Preparation and Validation of the Animal Models

**Figure 2:**
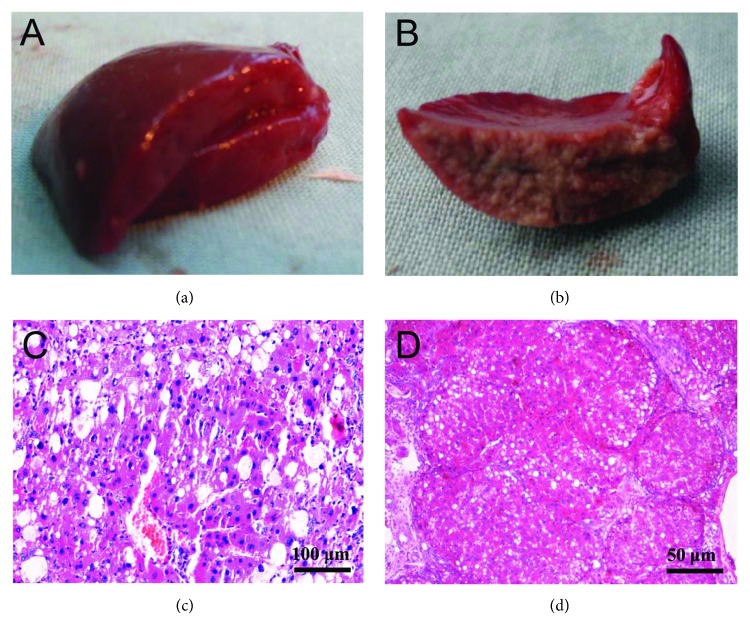
Validation of the rat models with liver cirrhosis induced by a combination of alcohol and CCl_4_. The male Wister rats were treated with a combination of alcohol and CCl_4_ diluted in olive oil as described in the Materials and Methods. After 5 weeks, liver cirrhosis in the rats was produced and validated. (a) Normal liver in rats; (b) cirrhotic liver in rats; (c) representative image of hematoxylin-eosin (H&E) stained liver section after liver cirrhosis was induced by a combination of alcohol and CCl_4_ for 9 weeks; and (d) representative image of H&E stained liver section after liver cirrhosis was induced by a combination of alcohol and CCl_4_ for 11 weeks.

After the male Wister rats were treated with a combination of alcohol and CCl_4_ for 12 weeks, the presence of liver cirrhosis in the rats was evidenced by histological examination of the liver tissue sections, exhibiting a characteristic pattern: disappearance of the hepatic lobule structure, inflammatory cell infiltration around the portal areas, formation of the fibrous cord, absence of the central portal vein, and formation of false lobules (Figure 2). Next, the rats with liver cirrhosis were subjected to three different techniques for hepatic vascular exclusion of liver surgery: Pringle maneuver in the PR group, hemihepatic vascular occlusion in the HH group, or hepatic blood inflow occlusion without hemihepatic artery control in the WH group. A total of 96 rats survived the surgery with success rates of 97% for the HH group and 100% for the WH group, which were higher than the rate of 94.1% in the PR group. The subsequent morphology examination confirmed the successful preparation of the rat models for this study.

### 3.2. Effects of the Different Techniques for Hepatic Vascular Exclusion during Resection on Liver Function after Hepatic I/R in Rats

**Figure 3:**
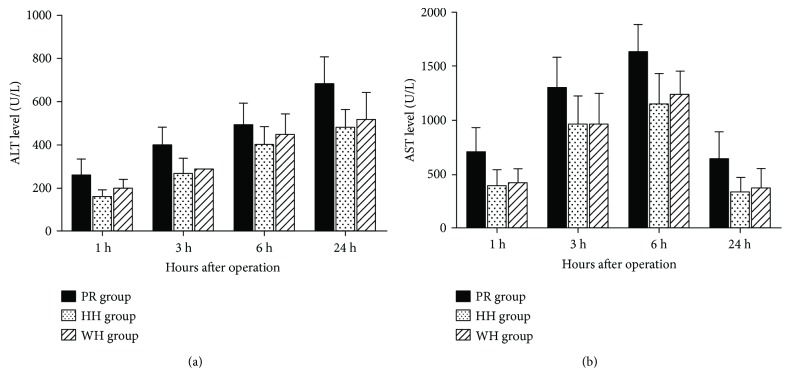
Effects of different techniques for hepatic vascular exclusion during resection on the liver enzymes ALT and AST after hepatic I/R in rats. After the rats in each group underwent resection with the three different techniques for hepatic vascular occlusion, the liver blood supply was restored and the levels of liver enzymes ALT and AST in serum samples from the rats were examined as described in the Materials and Methods. (a) ALT and (b) AST levels in the PR group, HH group, and WH group. ALT and AST levels in the PR group were significantly higher than those in the HH and WH groups (*P* < 0.05).

After the liver blood supply was reperfused in the experimental rats, the liver pathology and the liver enzyme ALT and AST levels were examined to detect inflammation and damage to the rat liver in the different groups undergoing the three techniques for hepatic vascular exclusion during hepatic surgery. After reperfusion was performed at each time point, the pathological morphology was examined with an inverted microscopy, which revealed more severe pathology in the PR group than in the WH and HH groups, while there was no significant difference in the pathological changes between the WH and HH groups. In addition, after the liver blood supply was recovered at each time point, ALT and AST levels in the PR group were significantly higher than those in the WH and HH groups (*P* < 0.05), while the difference was not statistically significant between the WH group and HH group (Figure 3). The morphology examination and the results of the liver enzyme ALT/AST tests indicated that hemihepatic vascular occlusion and hepatic blood inflow occlusion without hemihepatic artery control showed similar capability of reducing the hepatic I/R injury, but were better than the Pringle maneuver.

### 3.3. Effects of Different Techniques for Hepatic Vascular Exclusion during Resection on Inflammation after Hepatic I/R in Rats

**Figure 4:**
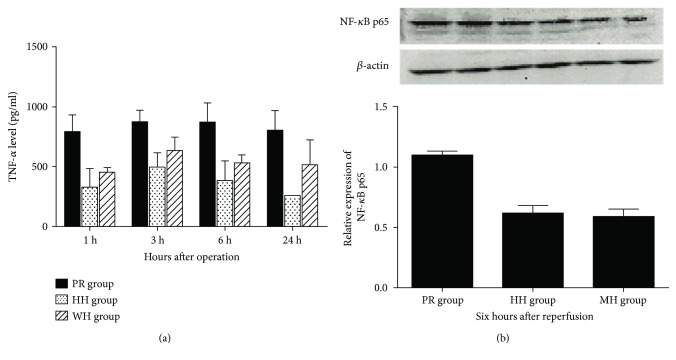
Effects of different techniques for hepatic vascular exclusion during resection on TNF-*α* and NF-*κ*B protein levels after hepatic I/R in rats. (a) TNF-*α* levels in the PR, HH, and WH groups. TNF-*α* levels in the PR group were significantly higher than those in the HH and WH groups (*P* < 0.05); (b) protein levels of NF-*κ*B. The NF-*κ*B protein level reached a peak at 6 h after reperfusion in all three groups, and NF-*κ*B levels at this time point in the HH and WH groups were significantly lower than those in the PR group (*P* < 0.05), but did not differ significantly from each other.

Hepatic I/R in rats apparently led to inflammation and damage, and the TNF-*α*/NF-*κ*B signaling pathway is known to be involved in the regulation of inflammation and other biological processes. Therefore, we examined the effects of the three techniques for hepatic vascular exclusion during resection on levels of TNF-*α*, a proinflammatory cytokine, and NF-*κ*B, a well-accepted transcription factor associated with inflammation after hepatic I/R in rats. As shown in Figure 4, the levels of TNF-*α* in the PR group were significantly higher than those in the WH and HH groups (*P* < 0.05), whereas there was no significant difference between the WH and HH groups. The protein levels of NF-*κ*B (p65) reached a peak at 6 h after reperfusion in all three groups, and NF-*κ*B (p65) protein levels at this time point in the WH and HH groups were significantly lower than those in the PR group (*P* < 0.05) with no significant difference between the WH and HH groups.

**Figure 5:**
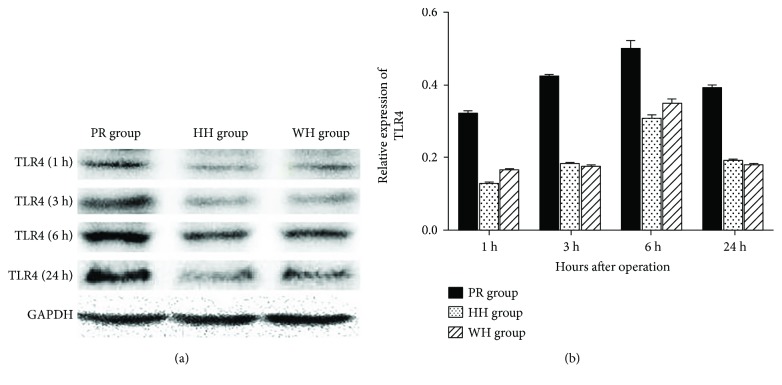
Effects of different techniques for hepatic vascular exclusion during resection on TLR4 protein levels after hepatic I/R in rats. WB analysis was performed to determine protein levels of TLR-4 and GAPDH in the rat liver tissues. The levels of TLR4 protein were normalized to GAPDH levels as described in the Materials and Methods. (a) WB analysis of TLR4 protein levels at different time points (1, 3, 6, and 24 h) after reperfusion in the PR, HH, and WH groups; (b) normalized TLR4 protein levels at different time points (1, 3, 6, and 24 h) after reperfusion in the PR, HH, and WH groups. TLR4 protein levels in the PR group were significantly higher than those in the HH and WH groups (*P* < 0.05), whereas there was no significant difference between the HH and WH groups.

**Figure 6:**
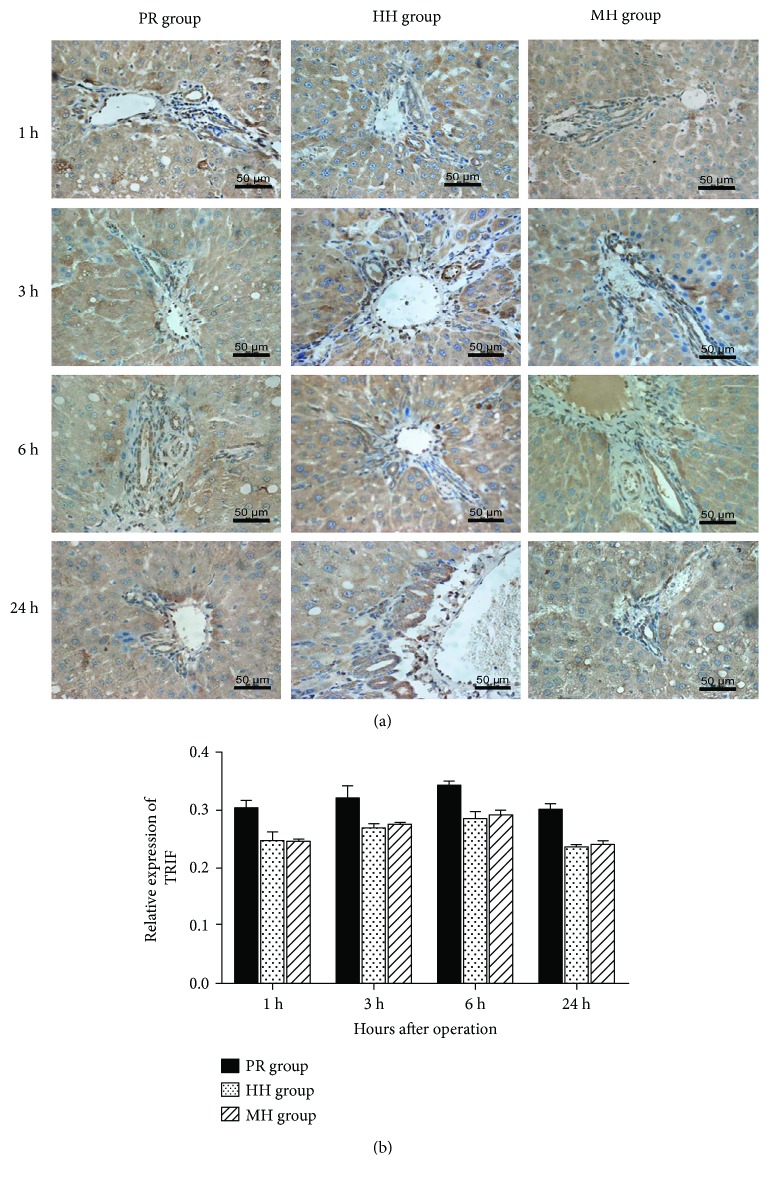
Effects of different techniques for hepatic vascular exclusion during resection on TRIF expression after hepatic I/R in rats. TRIF protein expression was determined by immunohistochemistry at different time points (1, 3, 6, and 24 h) after reperfusion in the PR, HH, and WH groups. (a) Representative images of immuno histochemical staining of TRIF in the liver sections from rats in the PR, HH, and WH groups; (b) quantification of IRIF expression in the liver of rats in the PR, HH, and WH groups. The TRIF protein level was significantly higher in the PR group than in the HH and WH groups (*P* < 0.05), while the difference between HH group and WH group was not statistically significant.

We asked whether the TRIF-dependent pathway, in which TLR signaling regulates expression of a wide range of genes that orchestrate the inflammatory response, contributes to the differential effects of the three techniques for hepatic vascular exclusion during resection on hepatic inflammation. Protein levels of both TRIF and TLR4 were determined in rat liver tissues from each group. As shown in Figures 5 and 6, at each time point, TLR4 and TRIF expression levels in the WH and HH groups were significantly lower than those in the PR group (*P* < 0.05), while the differences between the WH and HH groups were not statistically significant, suggesting that less prominent activation of the TLR4/TRIF transduction pathway may contribute to the reduced hepatic I/R injury that occurred in the two groups of blood inflow occlusion without hemihepatic artery control and hemihepatic vascular occlusion compared with the Pringle maneuver group.

### 3.4. Effects of Different Techniques for Hepatic Vascular Exclusion during Resection on HMOX1 Gene Expression after Hepatic I/R in Rats

**Figure 7:**
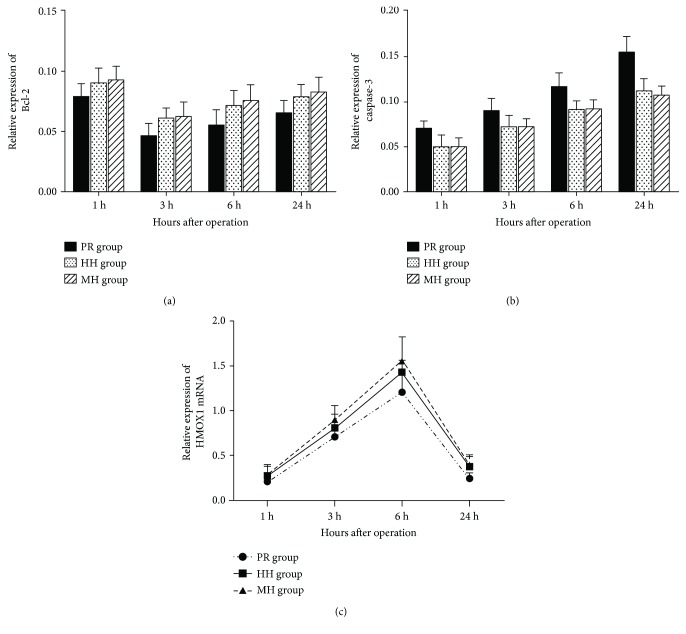
Effects of different techniques for hepatic vascular exclusion during resection on Bcl-2, caspase-3, and HMOX1 expression after hepatic I/R in rats. (a) Bcl-2 protein expression in the PR, HH, and WH groups. Bcl-2 protein levels were significantly lower in the PR group than in the HH and WH groups (*P* < 0.05); (b) caspase-3 protein expression in the PR, HH, and WH groups. Caspase-3 protein levels were significantly higher in the PR group than in the HH and WH groups (*P* < 0.05). (c) HMOX1 mRNA expression was determined by real-time qRT-PCR as described in the Materials and Methods. HMOX1 gene expression was significantly higher in the HH and WH groups compared with the PR group and reached a peak value at 6 h after reperfusion (*P* < 0.05).

In addition to inflammation, apoptosis is another central mechanism leading to hepatic damage in I/R injury. Bcl-2 protein, an apoptosis regulator, can protect cells from undergoing apoptosis by preventing a caspase-3-dependent proteolytic cascade. The protein levels of Bcl-2 decreased to the lowest value at the 3 h time point and were lower in the PR group than in the HH and WH groups (*P* < 0.05). Caspase-3 protein expression was continuously increased after reperfusion, and it was higher in the PR group than in the HH and WH groups (*P* < 0.05). These results indicate that hepatic blood inflow occlusion without hemihepatic artery control can reduce hepatic I/R injury by reducing the levels of caspase-3 protein, but increasing the level of Bcl-2 protein, thereby reducing apoptosis (Figures 7(a) and 7(b)). Since HMOX1 has been found to exert antiapoptotic properties, protecting cells against apoptosis, we assume that HMOX1 may contribute to the lower numbers of cells undergoing apoptosis in the HH and WH groups compared with the PR group. Next, the HMOX1 mRNA expression was determined by real-time qRT-PCR in the rat liver tissues. As shown in Figure 7(c), HMOX1 gene expression was significantly greater in the HH and WH groups compared with the PR group and reached a peak value at 6 h after reperfusion (*P* < 0.05).

## 4. Discussion

Hepatic I/R injury, as a major cause of liver damage and even liver failure, is a serious concern after hepatectomy, in particular, after resection was performed in the cirrhotic liver. Therefore, approaches to reduce harm to the liver and enhance hepatic protection are of significant importance. In the this study, we directly compared the benefits and harms of the three different surgical techniques for hepatic vascular exclusion, the Pringle maneuver, hemihepatic vascular occlusion, and hepatic blood inflow occlusion without hemihepatic artery control, during resection in the cirrhotic liver and their underlying molecular mechanisms using a rat model of liver cirrhosis. The main findings were as follows: (1) all three techniques for hepatic vascular exclusion were safe, with the rats that underwent hemihepatic vascular occlusion and hepatic blood inflow occlusion without hemihepatic artery control during resection responding to the hepatic I/R injury similarly but better than those that underwent surgery with the Pringle maneuver (Figure 3); (2) hepatic I/R inflammation was less severe in rats subjected to blood inflow occlusion without hemihepatic artery control and hemihepatic vascular occlusion compared with those subjected to the Pringle maneuver through, at least in part, the TNF-*α*/NF-*κ*B signaling and TLR4/TRIF transduction pathways (Figures 4–6); and (3) direct comparison of HMOX1 expression showed similar levels between the HH and WH groups and significantly lower levels in the Pringle maneuver group (Figure 7).

Of the dual blood supply of the normal liver, 70–75% is derived from the portal vein, while the remaining blood flow is through the hepatic arteries, which provides 40–60% of the oxygen, and is capable of flowing to the entire vascular network of the liver with a high-pressure gradient. During portal vein occlusion, the oxygen consumption of the liver maintains the prior level, suggesting that the hepatic arteries alone can supply the liver with sufficient oxygen. In our study, the histopathological changes in hepatic ischemia and reperfusion injury seemed to be minimal in both HH and WH groups, which were mainly attributed to the preservation of both the contralateral hemihepatic arterial blood supply and oxygen supply.

Recent decades have witnessed rapid progress in our understanding of inflammation and the involved molecular pathways, which include but are not limited to the TNF-*α*/NF-*κ*B signaling and TLR4/TRIF transduction pathways. In the TNF-*α*/NF-*κ*B signaling pathway, TNF-*α*, a proinflammatory cytokine, can trigger the dissociation between NF-*κ*B and its inhibitor I-*κ*B, which can induce the activation of NF-*κ*B, a transcription regulator [18]. Known as a master swatch, NF-*κ*B initiates downstream gene regulation associated with a wide range of cellular processes. In the TLR4/TRIF transduction pathway, TLR4 signaling via TRIF is essential for late activation of NF-*κ*B and is critical for the inflammatory response [21, 22]. In the present study, we found that TNF-*α* and NF-*κ*B levels were significantly lower in the WH and HH groups compared with the PR group, which may explain, at least in part, our observation that rats in the WH and HH groups had better responses to the inflammation induced by hepatic I/R in comparison with those in the PR group. Hepatic blood inflow occlusion without hemihepatic artery control can guarantee oxygen provision for the left liver, reduce activation of NF-*κ*B and expression of inflammatory factor TNF-*α* in the liver, and thereby decrease inflammation. This seems to be one of the mechanisms by which hepatic I/R injury was reduced.

Liver resection performed for HCC in patients with liver cirrhosis can be challenging, as hepatic cirrhosis largely increases the risk of liver failure and other adverse outcomes after hepatectomy. Therefore, enhancement of cytoprotective effects to preserve better liver function could improve the prognosis of patients undergoing liver resection. HMOX1 is a cytoprotective enzyme that catalyzes hepatic heme breakdown to produce biliverdin, carbon monoxide (CO), and ferrous iron. In fact, hepatic HMOX1 has been widely studied, and a wealth of scientific evidence has shown that HMOX1, mainly through a mammalian transcription factor Bach1, can protect cells from oxidative stress and apoptosis [23–26]. In addition, the anti-inflammatory activities of HMOX1 are well documented, and it is reported to protect hepatocytes from reperfusion injury. In our studies, the direct comparison of HMOX1 mRNA expression by real-time quantitative RT-PCR showed that HMOX1 expression was significantly greater in the HH and WH groups compared with the PR group. Based on the antiapoptotic activity of HMOX1, we assumed that enhanced levels of HMOX1 in the HH and WH groups likely contributed to the lower numbers of apoptotic cells in these groups. Furthermore, upregulation of HMOX1 is worth considering as an effective approach to better protect hepatocytes, which may also lead to better clinical outcomes after liver resection in HCC patients with liver cirrhosis.

Our study has some limitations. Although a growing body of research has been conducted to understand the inflammatory response and its molecular mechanisms when the human body is confronted by various stresses, our knowledge about this complex process is still in its infancy. Among the vast number of chemokines and signaling pathways involved in the inflammatory response, the TNF-*α*/NF-*κ*B signaling and TLR4/TRIF transduction pathways were studied in this present study. More insightful studies are needed to identify the molecular mechanisms that are responsible for the important outcomes of hemihepatic vascular occlusion and hepatic blood inflow occlusion without hemihepatic artery control during resection compared with the Pringle maneuver in rats. In addition, animal models cannot provide the exact conditions of liver cirrhosis and resection in humans and thus, have a number of limitations as well. Studies using human liver and blood samples are underway in our laboratory.

In conclusion, both hemihepatic vascular occlusion and hepatic blood inflow occlusion without hemihepatic artery control confer better preservation of liver function and better protection against I/R injury than the Pringle maneuver during liver resection in cirrhotic rats by differentially impacting, at least in part, the TNF-*α*/NF-*κ*B signaling and TLR4/TRIF transduction pathways and HMOX1 expression. Our results also suggest that upregulation of HMOX1 may provide better protection of hepatocytes and lead to better clinical outcomes after liver resection in HCC patients with liver cirrhosis.
